# No Distinct Cytokine, Chemokine, and Growth Factor Blood Profile Associated With Monkeypox Virus Clade IIb Infected Patients

**DOI:** 10.1002/jmv.70320

**Published:** 2025-03-29

**Authors:** Eugene Bangwen, Nicole Berens‐Riha, Nicky de Vrij, Ann Ceulemans, Isabel Brosius, Elise De Vos, Thao‐Thy Pham, Emmanuel Bottieau, Johan van Griensven, Palmer Masumbe Netongo, Marjan Van Esbroeck, Koen Vercauteren, Christophe Van Dijck, Wim Adriaensen, Laurens Liesenborghs

**Affiliations:** ^1^ Department of Clinical Sciences Institute of Tropical Medicine Antwerp Belgium; ^2^ Virology, Antiviral Drug & Vaccine Research Group, Department of Microbiology, Immunology and Transplantation KU Leuven Leuven Belgium; ^3^ Adrem Data Lab, Department of Computer Science University of Antwerp Antwerp Belgium; ^4^ Department of Biomedical Sciences Institute of Tropical Medicine Antwerp Belgium; ^5^ Molecular Diagnostics Research Group Biotechnology Centre‐University of Yaounde I (BTC‐UYI) Yaounde Cameroon; ^6^ Biology Program, School of Science Navajo Technical University Crownpoint New Mexico USA

**Keywords:** cytokines, immunology of mpox, monkeypox virus, mpox

## Abstract

Previous studies indicated Clade I monkeypox virus infection to be associated with marked elevation of proinflammatory cytokines. This remains unexplored for Clade II‐associated disease, which has different clinical manifestations and prognosis. We used a 65‐plex cytokine, chemokine, and growth factor (CCG) panel to analyze serum samples of 100 male acute Clade IIb mpox patients and 26 healthy controls in Belgium. Cluster analyses revealed no strong or distinct CCG profiles distinguishing mpox patients from controls but suggested trends in certain cytokine modulation. Individual CCG analyses found elevated levels of cytokines (MIF, CD30, IL2R, IL18, APRIL, and TNFRII), chemokines (CCL4, CCL8, CCL22, CCL24, CXCL9, CXCL10, CXCL11, CXCL12, and CXCL13), and growth factors (HGF and VEGFA) in patients, while CCL11 and CXCL5 were significantly suppressed. We detected no differences in key proinflammatory cytokines, IL‐1α, IL‐1β, IL‐6, IL‐8 or anti‐inflammatory cytokines, IL‐4, IL‐10, IL‐13. In patients living with HIV, comparison with pre‐outbreak samples showed an increase in CXCL13 and a decrease in CXCL5, CCL2, CCL24, HGF, SCF, and TWEAK. The absence of discriminatory CCG profiles in Clade IIb mpox patients compared to healthy controls suggests there may be limited clinical applications of those markers.

## 1 Introduction

Mpox is a viral disease caused by the monkeypox virus (MPXV). There are two genetically different clades known to cause disease in humans. Clade I MPXV (formerly known as the Congo Basin Clade), recently subdivided into Ia and Ib, is endemic in Central Africa and has been reported to cause more severe disease with a case fatality ranging from 1% to 10% [1, 2]. Clade II, further subdivided into Clades IIa and IIb, originated from West Africa and has been associated with less severe disease.

In 2022, sexual transmission of Clade IIb MPXV triggered a global outbreak, leading to over 90 000 cases worldwide and disproportionately affecting men who have sex with men [3]. Compared to previously reported Clades I and II outbreaks on the African continent, most Clade IIb infections during the global outbreak were relatively mild. Depending on the country, between 1% and 13% of patients required hospitalization and, overall, 115 deaths (< 0.01% of patients) were registered [3]. From published studies, between 36% and 42% of patients were people living with the human immunodeficiency virus (HIV) [4, 5], although it is unknown whether the HIV affects the risk of acquiring mpox. Uncontrolled HIV, on the other hand, is a known risk factor for severe mpox disease [4].

Individuals infected with Clade IIb MPXV typically present with nonspecific prodromes such as fever, fatigue, or myalgia, followed by a characteristic mpox rash, which can be localized at the site of infection or more generalized. Often, the rash is accompanied by lymphadenopathies and signs of mucosal infections such as proctitis, urethritis, or pharyngitis [2, 5]. Life‐threatening complications, such as necrotizing skin lesions, lung involvement, sepsis, and multiple organ failure, are rare and generally occur primarily in severely immunocompromised individuals, such as those with uncontrolled HIV infection [6].

Cytokines are recognized for their strong potential as biomarkers of disease progression as well as in immunotherapeutic applications. Currently, no specific therapy has been proven to affect the outcome of mpox patients. Several antiviral drug candidates are under investigation, but the development of drug resistance is of concern [7, 8]. An alternative strategy is to target the immune system and try to dampen the proinflammatory immune response, a strategy that has proven to be successful in the treatment of other viral diseases, for example, through the use of corticosteroids or inhibition of IL‐6 in COVID‐19 [9, 10, 11].

However, the inflammatory response to MPXV infection is not well characterized. Existing knowledge stems from studies on related orthopoxviruses, derived from ex vivo or in vitro studies [12]. Only three other studies with small sample sizes and limited cytokine panels have evaluated the expression of peripheral blood cytokines, chemokines, and growth factors (CCG) associated with MPXV Clades I and IIb infection in humans. In a study with 19 Clade I MPXV‐infected patients in the Democratic Republic of the Congo (DRC), the authors observed an overproduction of both pro‐ and anti‐inflammatory cytokines which positively correlated with disease severity [13]. Two additional studies focused on cytokine expression, one after stimulation of isolated peripheral blood mononuclear cells (PBMCs) from 17 Clade IIb MPXV‐infected patients in Italy [14], and the other in 39 hospitalized mpox patients in China, presumably infected with Clade IIb [15]. However, results were not correlated with disease severity and the limited cytokine panels in both studies did not reveal a distinct CCG expression pattern.

To better understand the CCG expression patterns of Clade IIb mpox disease, identify potential biomarkers of disease progression, and explore possible immunotherapeutic targets, we characterized the expression of 65 CCGs in individuals with acute Clade IIb mpox, compared to healthy controls.

## 2 Material and Methods

### 2.1 Study Design and Selection of Participant Samples

To characterize the CCG response, we analyzed serum samples of PCR‐confirmed mpox patients, included in a prospective clinical mpox registry between May 23 and September 20, 2022. Data on demographics, exposure history, clinical signs, and symptoms were captured in a REDCap database, while biological samples were collected and stored in a biobank [5]. In this single‐center study, 162 patients provided written informed consent to use their data and samples for scientific research. From these, we had sufficient serum samples for 101 individuals.

As controls, we selected biobanked serum samples from 30 healthy volunteers. Additionally, in a subset of 10 mpox patients living with HIV, who had been monitored prior to the outbreak and had stored samples collected before contracting mpox, we selected one pre‐outbreak sample from each patient to allow for within‐patient CCG comparisons before and after the disease.

In a hospitalized mpox patient who had advanced HIV and lymphoma, we analyzed longitudinally collected serum samples at different time points (Days 7, 36, and 50).

### 2.2 Laboratory Procedures

CCGs were measured using the Immune Monitoring 65‐Plex Human ProcartaPlex Panel (Thermofisher Scientific, Massachusetts, USA). Frozen serum samples were thawed on ice, vortexed to homogenize, centrifuged at 10 000*g* for 10 min. Lyophilized standard mix for generation of standard curves, low and high controls supplied with the kit were diluted following the assay protocol and kept on ice. The capture bead mix, diluted standards, controls, samples, and other provided reagents were added following the manufacturer's instructions. All samples and controls were run in duplicates. Plates were run on a Luminex Bio‐Rad Bio‐Plex 200 which provided the fluorescent intensities per analyte for every well. The plate run results were uploaded to the ThermoFisher ProcartaPlex cloud app to generate standard curves and final concentrations per analyte.

### 2.3 Data Processing and Analyses

Prior to the final analysis, we excluded one patient sample and four controls due to sampling, pipetting error, or duplicate discordance in ProcartaPlex assay.

The CCG concentration data per plate exported from the ProcartaPlex cloud app were merged using the R statistical software [16]. To identify patterns within the CCG expression data, the CCG concentrations were log2‐normalized and subsequently standardized to *Z*‐scores. The resulting *Z*‐scores were then visualized in a heatmap generated with the package ComplexHeatmap v2.10.0 in the programming language R 4.1.1 [17]. The heatmap rows and/or columns were unsupervised hierarchically clustered using the “ward.D” cluster method. In addition, a summary heatmap of mpox+ and mpox− participants was created using only the per‐group median of the resulting *Z*‐scores. Next, to validate whether mpox patients, HIV‐infected mpox patients, and healthy participants would cluster distinctly based on their CCG expression, we applied principal component analysis (PCA) on the log2‐normalized and *Z*‐scored data, followed by *k*‐means clustering with *k* = 3.

Clinical variables used in the analyses were lesion counts (categorized: 0, 1–4, 5–25, 26–100, > 100), HIV status, and presence or absence of systemic symptoms (reported history of fever or fever measured at presentation, shivering, fatigue, generalized lymphadenopathy, headache, muscle, or joint pain), ongoing fever measured at presentation (≥ 38°C) and clinical proctitis (rectal pain, no confirmation by proctoscopy). Data on smallpox vaccination during childhood were collected. However, due to the incomplete nature of these data and the fact that several patients could not remember if they were vaccinated, we considered individuals born before 1977 to be vaccinated and those born in or after 1976 to be unvaccinated, based on the cessation of smallpox vaccination in Belgium.

Plots to show comparisons between individually selected CCGs were generated using GraphPad Prism 10.1.2 (GraphPad Software, La Jolla, CA). Statistical analyses were conducted using Stata (version 14.0, StataCorp, USA). Linear or logistic regression was employed to examine associations between cytokine expression and number of lesions or categorized clinical symptoms, respectively. We tested for possible interaction with age, sex, and HIV status in a linear regression model and applied the Mantel–Haenszel method for stratification if indicated. The Wilcoxon rank‐sum (Mann–Whitney *U*) test was applied for independent samples. A level of significance of 0.05 was used. We corrected for multiple testing using the Benjamini–Hochberg method with a false discovery rate of 0.1 [18].

## 3 Results

### 3.1 Demographic and Clinical Characteristics of Study Participants

For the final analysis, 136 samples from 126 individuals were included: 80 HIV‐negative mpox patients, 20 mpox patients living with HIV, 26 healthy controls, and 10 people living with HIV (PLWH) without mpox. All included mpox patients were male, with a median age of 40 years (IQR: 33–46). All of the PLWH were on antiretroviral treatment, had an undetectable viral load (all but 1 for whom viral load was unknown), and had a CD4 cell count above 400 cells/mm^3^ at the time they presented with mpox disease. Twenty‐five individuals were born before 1977 and thus most probably vaccinated against smallpox during their childhood. Most patients (77/100) presented with one or more systemic symptoms like (history of) fever, chills, fatigue, and generalized muscle or joint pain. Thirty‐five had acute fever at presentation, and 22 suffered from proctitis. Five patients had no skin lesions (only mucosal infection), 36 had between 1 and 4 lesions, 45 had between 5 and 25 lesions, and 12 presented 26 to 100 lesions. Only two patients had more than 100 lesions. Disease severity was, in general, low, as only two patients were hospitalized, and one died (Table 1).

**Table 1:** Demographic and clinical characteristics of mpox cases and healthy volunteers.

Characteristics	All mpox patients *n*/*N* (%) *N* = 100	Mpox PLWH *n*/*N* (%) *N* = 20	Mpox HIV‐negative *n*/*N* (%) *N* = 80	Healthy volunteers *n*/*N* (%) *N* = 26
Age in years *median (IQR)*	40 (33; 46)	44 (33; 54)	40 (33; 44)	38 (28; 51)
*Sex*				
Female	0	0	0	16/30 (66.7)
Male	100/100	20/20 (100.0)	80/80 (100.0)	10/30 (33.3)
*Clinical symptoms*				
Lesions				NA
0	5/100 (5.0)	0	5/80 (6.2)	
1–4	36/100 (36.0)	8/20 (40.0)	28/80 (35.0)	
5–25	45/100 (45.0)	9/20 (45.0)	36/80 (45.0)	
26–100	12/100 (12.0)	2/20 (10.0)	10/80 (12.5)	
> 100	2/100 (2.0)	1/20 (5.0)	1/80 (1.3)	
Proctitis	22/100 (22.0)	6/20 (30.0)	16/80 (20.0)	NA
Systemic symptoms	77/100 (77.0)	14/20 (70.0)	63/80 (78.8)	NA
Fever at presentation	35/100 (35.0)	6/20 (30.0)	29/80 (36.3)	NA
Born before 1977	25/100 (25.0)	8/20 (40.0)	17/80 (21.3)	8/26 (30.8)

### 3.2 Cluster Analysis Reveals Weak Discriminatory Power in Measured CCGs to Discern mpox Disease or Severity

**Figure 1:**
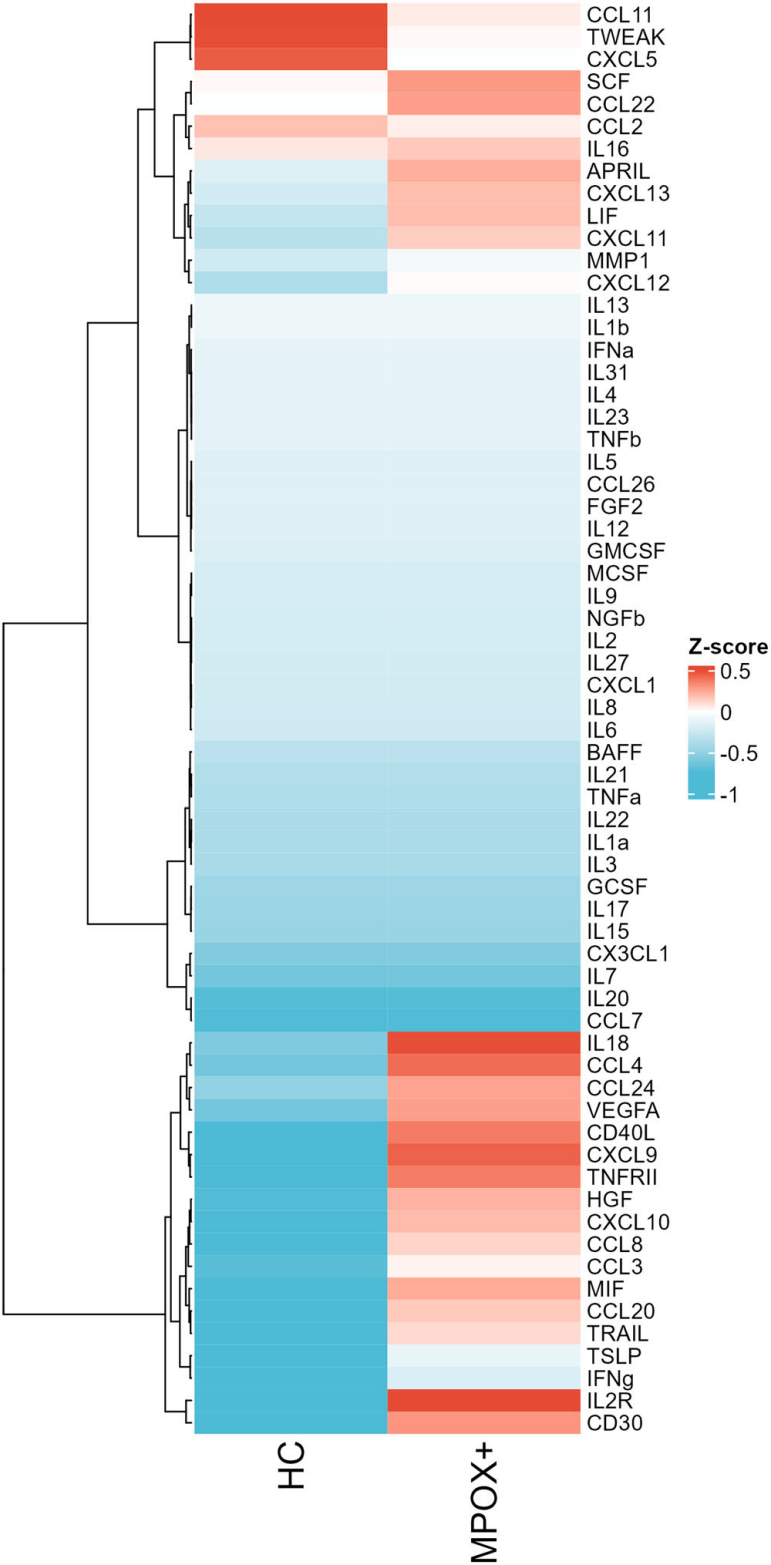
Heatmap displaying the within‐group median log2‐normalized *Z*‐scored CCG expression for acutely ill mpox patients versus healthy volunteers.

**Figure 2:**
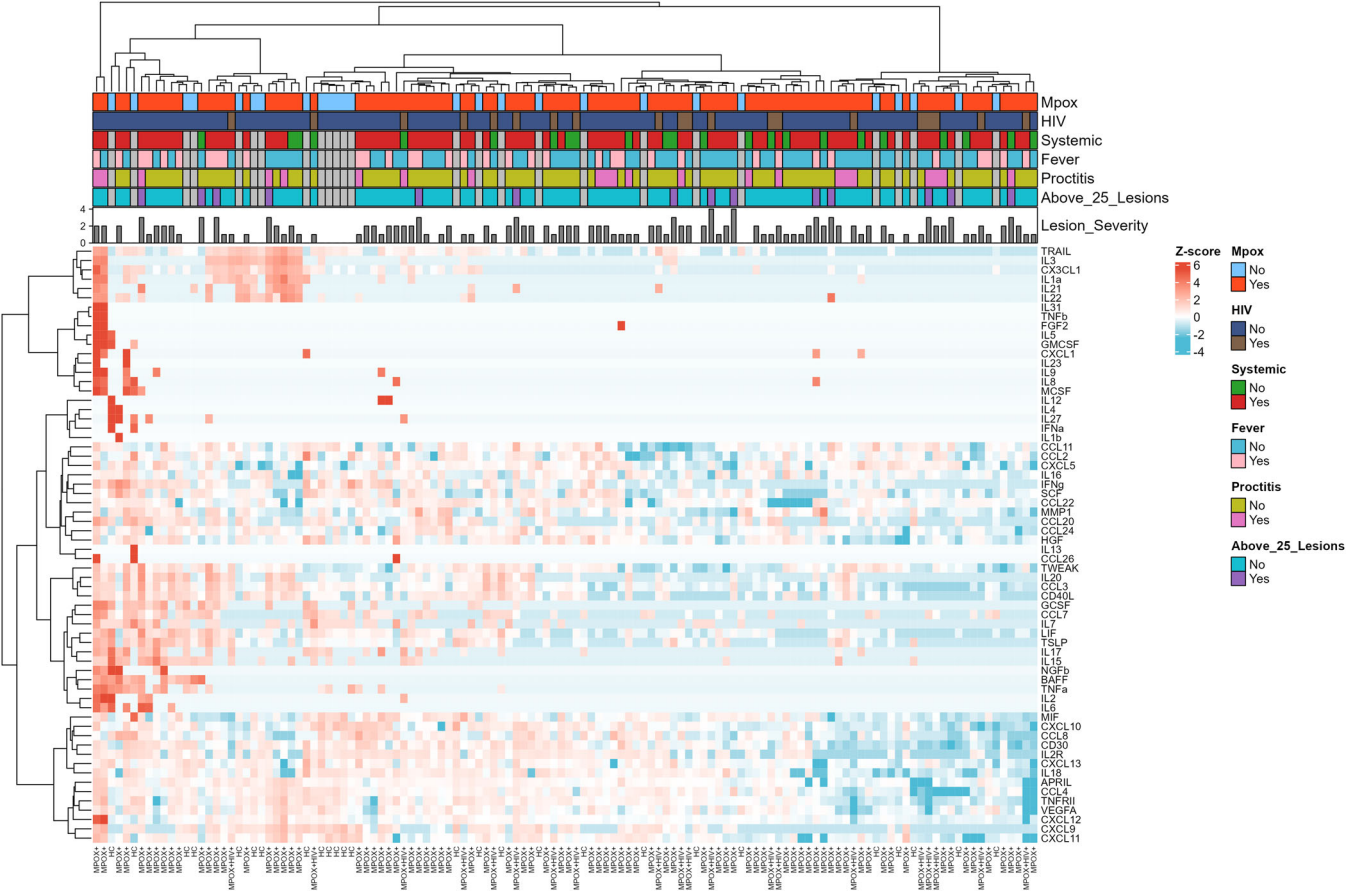
*Z*‐scored heatmap of CCG values, as well as clinical characteristics of each participant.

In a heatmap displaying the within‐group median log2‐normalized *Z*‐scored CCG expression for acutely ill mpox patients versus healthy volunteers, a distinct pattern of 20 different CCGs was observed, differentiating the two groups (Figure 1). However, when performing unsupervised hierarchical clustering on each participant's individual CCG expression, the clustering between mpox patients and controls was weak (Figure 2). A minority of patients and controls also did not cluster distinctly (*n* = 6 (23.3%) controls, *n* = 8 (8%) mpox patients). We observed no clear CCG patterns between PLWH and those not living with HIV, nor was there any observable correlation with the presence or absence of proctitis, fever, systemic symptoms, or lesion count.

**Figure 3:**
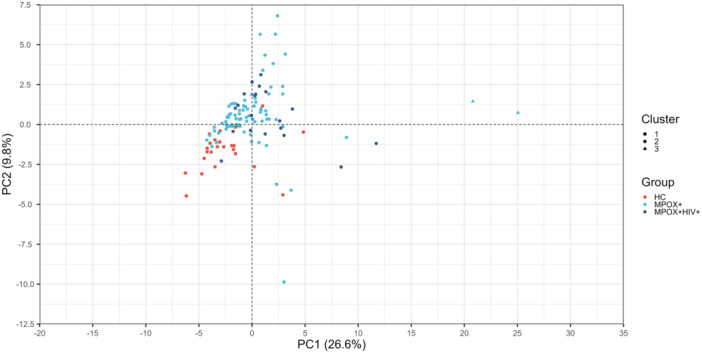
*K*‐means clustering following PCA on the log2‐normalized and *Z*‐scored CCG data to confirm the weak clustering observed with the heatmap.

To confirm the weak clustering observed in the heatmaps, we performed PCA and *k*‐means clustering on the log2‐normalized and *Z*‐scored CCG data (Figure 3). Here, we observed the same weak clustering, with the majority of mpox patients clustering distinctly from the healthy controls, although a minority did not. Together, this indicates a detectable alteration in the blood CCG profile of diseased patients, although not consistently across patients.

### 3.3 Clade IIb Patients Showed Assorted Nonspecific Expression of CCGs

**Figure 4:**
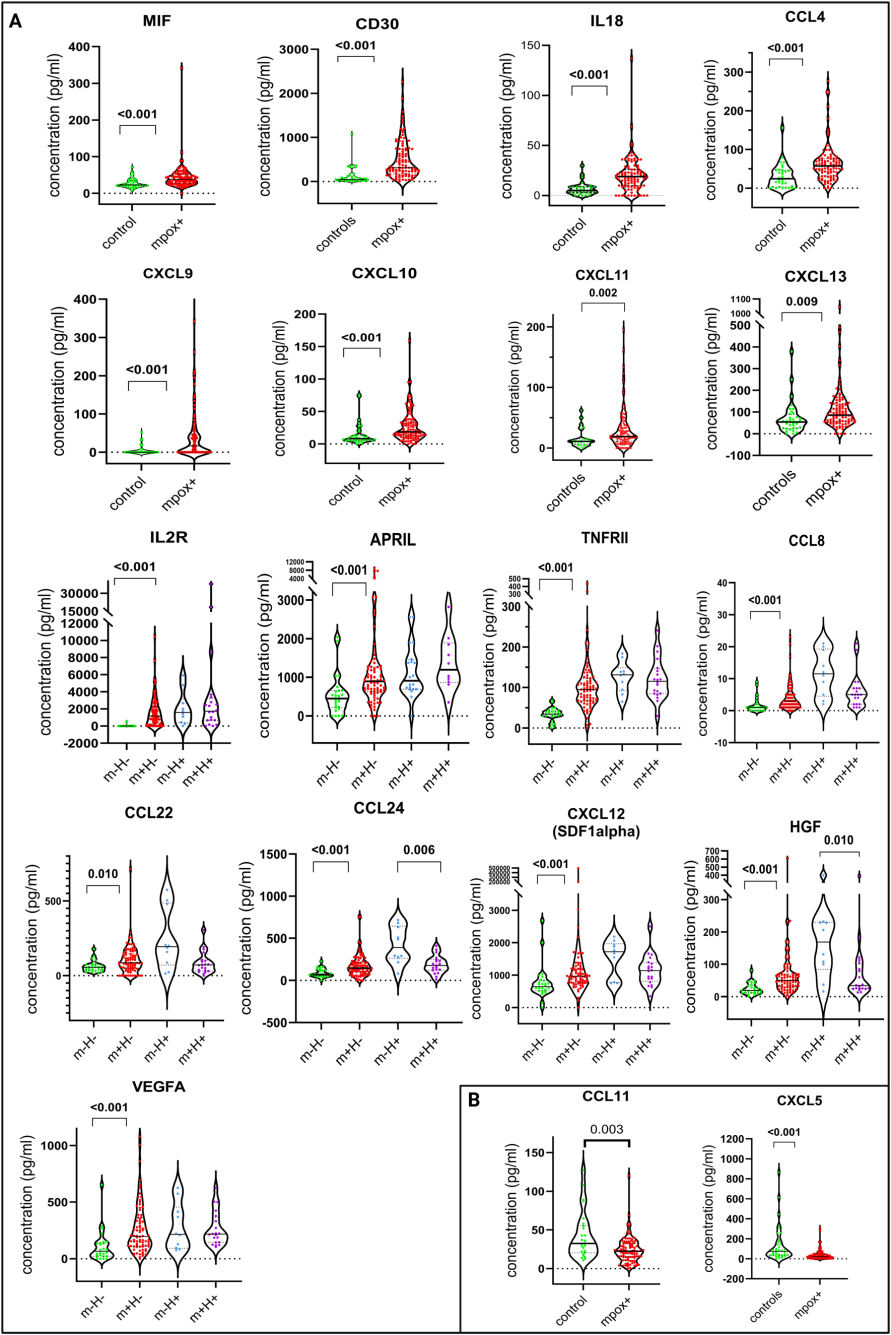
Comparison of the concentration (pg/mL) of individual CCGs in mpox patients (mpox+) and healthy participants (controls). In the case of interaction, we conducted a separate analysis stratified by HIV status. Mpox‐positive participants without HIV (m+H−, *n* = 80), healthy volunteers (m−H−, *n* = 26), mpox‐positive participants living with HIV (m+H+, *n* = 20), and available pre‐outbreak controls for HIV positive participants before mpox (m−H+, *n* = 10) Associations were tested by Mann–Whitney *U* test and *p* values reported. (A) Elevated CCG values in all mpox patients compared to healthy volunteers. (B) Suppressed CCG values in all mpox patients compared to healthy volunteers.

When comparing individual cytokine levels, we found that the following CCGs concentrations were significantly higher in mpox patients compared to healthy controls: cytokines (MIF, CD30, IL2R, IL18, APRIL, and TNFRII), chemokines (CCL4, CCL8, CCL22, CCL24, CXCL9, CXCL10, CXCL11, CXCL12, and CXCL13), and growth factors (HGF and VEGFA). In contrast, CCL11 and CXCL5 were found to be significantly more suppressed (Figure 4).

Interestingly, we did not detect a significant difference in the expression of key (and targetable) proinflammatory cytokines like IL‐1α, IL‐1β, IL‐6, IL‐8 or anti‐inflammatory cytokines like IL‐4, IL‐10, and IL‐13 between mpox patients and control volunteers.

#### 3.3.1 CCG Modulation Associated With HIV Infection

We observed a significant interaction between HIV status and mpox disease for the following CCGs: cytokines (APRIL, IL7, TWEAK, and TNFRII), chemokines (CXCL5, CXCL13, CCL2, CCL8, CCL22, CCL24, and CXCL12), and growth factors (HGF and SCF) (Supporting Information S3: Table 1). In the case of interaction, we conducted a separate analysis stratified by HIV status. Specifically, we compared: mpox‐positive participants without HIV (m+H−, *n* = 80) to healthy volunteers (m−H−, *n* = 26) and mpox‐positive participants living with HIV (m+H+, *n* = 20) to available pre‐outbreak controls (m−H+, *n* = 10).

For participants living without HIV compared to the healthy controls, (m+H−/m−H−) the CCGs APRIL, IL7, TNFRII, CXCL12, CXCL13, CCL8, CCL22, CCL24, and HGF were significantly increased, while CXCL5 was significantly decreased. No significant difference was observed for TWEAK, CCL2, and SCF. For participants living with HIV compared to pre‐outbreak controls (m+H+/m−H+), only CXCL13 showed a significant increase, whereas CXCL5, CCL2, CCL8, CCL22, CCL24, IL7, HGF, SCF, and TWEAK were significantly decreased. No significant difference was observed for APRIL, CXCL12, and TNFRII (Supporting Information S3: Table 1; Supporting Information S1: Figure 1 and Supporting Information S2: Figure 2).

#### 3.3.2 CCG Modulation Associated With Other Clinical Manifestations

**Figure 5:**
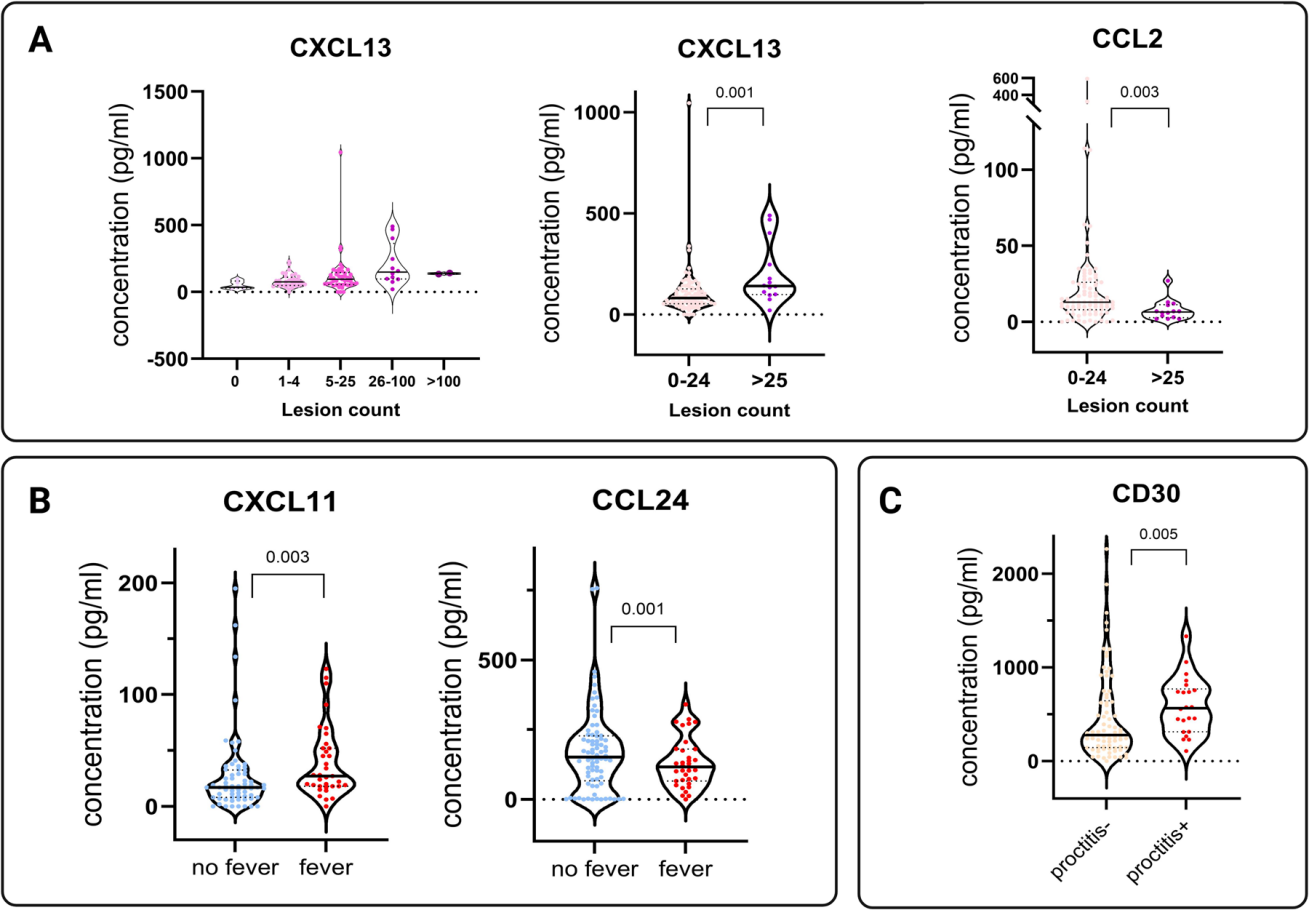
Comparison of the concentration (pg/mL) of individual CCGs in mpox patients with different clinical characteristics. Associations were tested by Mann–Whitney *U* test and *p* values reported. (A) Concentrations of individual CCGs in mpox patients with different lesion count categories. (B) Possible association of CXCL11 and CCL24 with the presence of ongoing fever during mpox disease. (C) Evidence of possible association of CD30 with proctitis during mpox disease.

When comparing cytokine expression in patients with different clinical manifestations within the mpox‐confirmed group, we found that the number of lesions were associated with higher levels of CXCL13 (*p* = 0.004, linear regression) and lower levels of CCL2. The presence of proctitis in mpox patients was significantly associated with an elevation of CD30 (*p* = 0.005). The presence of fever at admission was associated with an overexpression of CXCL11 (*p* = 0.003) as well as a suppressed expression of CCL24 (*p* = 0.001) (Figure 5).

The only mpox patient who died had general CCG elevations with extremely elevated IL2R values. Over a period of 6 weeks from initial diagnosis of mpox until death, his IL2R measurements were as follows: 37 804; 173 677; 202 355, compared to a median value of 0 in the healthy controls, 1500 in HIV+/mpox− and 2500 in HIV+/mpox+. Measurements at all three‐time points of all CCGs showed elevated and progressively rising values for many CCGs including the interleukins IL1alpha, IL2R, IL5, IL6, IL7, IL8, IL16, and IL18. No elevation was observed for TNFα, TNFβ, NGFβ, INFα, GM‐CSF, and the other interleukins. However, this patient was HIV‐infected and suffered from a lymphoma; it is likely that the mpox infection triggered the progression of his underlying disease.

## 4 Discussion

To broadly characterize the CCG response following mpox due to Clade IIb MPXV infection, we investigated the expression of 65 individual CCGs in the serum of 100 mpox patients (including 20 PLWH) and 26 healthy volunteers. Our findings revealed a rather mild and nondistinctive pattern of targeted CCGs in infected mpox patients compared to healthy volunteers, regardless of their clinical symptoms or HIV status.

In contrast to the strong and severity‐related CCG profile described among Clade I MPXV patients, our findings suggest that Clade IIb is not associated with the extensive production of proinflammatory cytokines. So far, only the study by Johnston and colleagues evaluated CCG concentrations in sera of 19 acutely ill mpox patients with Clade I infection, using a 30‐plex panel focused on possible association with disease severity [13]. In that study, they observed an upregulation of T helper (Th) 2‐associated cytokines IL‐4, IL‐6, IL‐5, and IL‐10 and attenuation of Th1‐associated cytokines, such as IFN‐α, IFN‐γ, TNF‐α, IL‐2, and IL‐12 in serum of infected patients in the DRC. In patients with severe disease, an excessive upregulation of cytokines indicative of a cytokine storm was observed. This markedly contrasts with our findings for Clade IIb infections where among the above‐mentioned CCGs, only IL‐2R was likewise significantly elevated. IFN‐α, IFN‐γ, TNF‐α, IL‐2, IL‐7, and IL‐12 which were not elevated in Clade I, equally showed no significant elevation. Additionally, given the mild nature of our Clade IIb patient cohort, GM‐CSF and IL‐10, which were not elevated in Clade I mpox cases, were similarly not elevated in our Clade IIb cohort (Table 2).

**Table 2:** Summary of cytokine expression in mpox across studies.

Cytokine	MPXV Clade I [13]	MPXV Clade IIb (PBMCs) [14]	MPXV Clade IIb (presumed) [15]	MPXV Clade IIb (study samples)
IL‐1RA	↑	—	↑	—
IL‐1β	↑	↑	↑^b^	n
IL‐2	n^a^	—	↑^b^	n
IL‐2R	↑ (↑↑ serious)	—	—	↑ (↑↑ PLWH)
IL‐4	↑	—	n	n
IL‐5	↑	—	n	n
IL‐6	↑ (↑↑ serious)	↑	↑^b^	n
IL‐7	n	—	n	n
IL‐8	↑	↑	↑^b^	n
IL‐10	↑ (↑↑ severe)	—	↑^b^	n
IL‐12	n^a^	—	—	n
IL‐13	↑	—	n	n
IL‐15	↑	—	↑^b^	n
IL‐17	↑	—	n	n
IL‐18	—	—	—	↑^c^
CCL2/MCP‐1	↑	—	n	n (↓ PLWH)
CCL3	—	—	—	↑
CCL4	—	—	—	↑
CCL5 (RANTES)	↑	—	↑	—
CCL8	—	—	—	↑^c^
CCL11 (Eotaxin)	n	—	n	↓
CCL22	—	—	—	↑^c^
CCL24	—	—	—	↑^c^ (↓ PLWH)
GM‐CSF	↑ (Severe > mild)	—	n	n
TNF‐α	n	↑	↑	n
TNF‐RII	—	—	—	↑^c^
INF‐α	n	—	—	n
IFN‐γ	n^a^	—	—	n
CXCL5	—	—	—	↓
CXCL9 (MIG)	n	—	—	↑^c^
CXCL10 (IP‐10)	n	—	↑	↑^c^
CXCL11	—	—	—	↑
CXCL12	—	—	—	↑
CXCL13	—	—	—	↑^c^
APRIL	—	—	—	↑
CD30	—	—	—	↑
MIF	—	—	—	↑^c^
HGF	—	—	—	↑^c^ (↓ PLWH)
SCF	—	—	—	↓ (PLWH)
VEGFA	—	—	n	↑

^a^ Within the normal range but interpreted as dampened by the authors.

^b^ Within the normal range/very low median values (pg/mL), few outliers.

^c^ Elevated compared to healthy controls, no normal range established.

The differences in CCGs expressed in mpox due to Clades I and IIb appear to correlate with the variations in disease presentation between infections caused by these two virus clades. Clade I has been historically reported to cause more severe disease compared to Clade II [1, 19, 20]. In our study, the lack of distinctive patterns in cytokine expression observed could be attributed to the fact that only a few patients exhibited severe disease, with one fatality. A common way of defining disease severity is the WHO classification, which is based on lesion count, with over 250 lesions indicating severe disease and 100–250 lesions indicating moderate disease. In our study, none of the patients had > 250 lesions, and only 2 of the 100 patients had over 100 lesions one of whom died. Only CXCL13 was positively correlated with lesion count but elevations were not distinct enough to be used as a clinical marker. In COVID‐19, CXCL13 was found to be one of the best predictors of disease severity and ICU admission [21]. CXCL13 is essential for guiding B cells to lymphoid tissues for antigen exposure [21, 22]. CD30, part of the tumor necrosis factor receptor family, contributes to cell proliferation of peripheral T‐cells, and its expression is mainly restricted to virus‐infected lymphocytes and neoplasms of lymphoid origin [23]. It was significantly, but not consistently, elevated in patients with proctitis, a condition caused by localized inflammation, which may limit systemic cytokine expression. The more severe bacterial superinfections developed mostly after the first presentation, by which time blood samples had already been collected, possibly also contributing to the differences in cytokine expression compared to Clade I. Unspecific systemic symptoms such as fever were highly prevalent during the disease but generally mild.

Baseline levels of some CCGs like APRIL (*p* = 0.001) or IL2R (*p* < 0.001) in the 10 PLWH before mpox were higher compared to the 26 healthy controls (Supporting Information S3: Table 1). During mpox, different effects could be observed in PLWH. Some cytokines showed similar levels to pre‐mpox (APRIL, IL‐2R, CXCL10), while CXCL13 was significantly elevated and others were even decreased (CXCL5, CCL24, HGF, CCL2, CCL22, and SCF). These differences warrant further investigation with a larger sample size, as the current study's limited sample size of PLWH restricts the scope for interpretation. The one patient with HIV and severe mpox who died had a lymphoma and it is likely that the mpox infection triggered the progression of his underlying disease. The other PLWH in this study experienced mild to moderate mpox. All were on antiretroviral therapy, and none had a CD4 count below 200. However, there was an observed interaction with HIV status in mpox patients for 22 cytokines. During HIV‐1 infection, secretion of Th type 1 cytokines, such as interleukin IL‐2 and IFN‐gamma, is in general rather decreased, whereas production of Th type 2 and proinflammatory cytokines like IL‐4, IL‐10 and IL‐1, IL‐6, IL‐8, TNF‐α and others is often increased [24]. This pattern could not be seen in our PLWHs, but it might be more pronounced in untreated patients.

Only two studies have explored CCG expression in mpox caused by Clade IIb MPXV infection. Agrati and colleagues examined cytokine expression in 17 Italian patients by stimulating PBMCs, revealing elevated levels of IL‐1β, IL‐6, IL‐8, TNF‐α, IFN‐γ, IL‐2, and TNF which remained high even after recovery [14]. However, ex vivo PBMC stimulation may not accurately represent the natural systemic CCG profile. A more recent longitudinal study from China assessed 27 CCGs in 39 hospitalized mpox patients (in isolation rather than based on clinical manifestations), finding increased levels of G‐CSF, IL‐1β, IL‐6, IL‐8, IL‐10, IP‐10, and RANTES compared to healthy controls [15]. HIV‐positive patients had higher levels of MIP‐1α, MIP‐1β, G‐CSF, IL‐4, and FGF‐basic. Despite these findings, many median cytokine levels were low or within the normal range, and no correction for multiple testing was reported. Given the limitations of PBMC stimulation in the first study and the small sample sizes in both, these results should be interpreted with caution.

In addition to the absence of discriminating patterns between patients and healthy controls in our study, no typical inflammatory pathways seemed enriched (e.g., interferon pathway) among the individual CCGs that were differentially expressed. In line, well‐established pro‐ (IL‐1, IL‐6, and TNF‐α) and anti‐inflammatory regulator cytokines (IL‐4, IL‐10, IL‐13) were not differentially expressed in the mpox patients. This absence of robust CCG profiles in peripheral blood suggests a limited value for patient management of Clade IIb mpox patients through interventions modulating these pathways or commonly targeted cytokines such as IL‐6 and IL‐10 as suggested by others [10, 11]

This is the only study that conducted a comprehensive screening of CCG profiles associated with acute Clade IIb mpox, investigating the expression of up to 65 CCGs in 100 patients in Belgium. The limitations, however, are the small number of controls, mostly female (> 60%) and the absence of longitudinal follow‐up. Furthermore, all mpox patients were acutely ill at the time of blood draw but as symptoms can last between a few days and 2–3 weeks, measurements were done when patients presented at the clinic, meaning at different time points during the acute disease. Proctitis, other bacterial superinfections in different stages and severity, and other partly unknown underlying factors might likewise influence the cytokine production and interact with the reactions to mpox. Finally, this was conducted as a retrospective, single‐center study.

## 5 Conclusions

Reflecting differences in clinical presentation, severity and mortality rate, Clades I and II MPXV infections seem to have different blood CCG profiles, with a less pronounced increase in inflammatory cytokines in Clade IIb compared to Clade I infections. The observed differences are likely multifactorial. While variations in virulence between Clades I and II may contribute to variations in CCG profiles [20], differences in the affected populations and access to healthcare likely contribute as well [25]. Unfortunately, studies directly comparing MPXV clades in humans are currently lacking. Despite some individual CCG changes in acutely ill mpox Clade IIb patients compared to healthy participants, the absence of discriminating CCG profiles or enriched pathways in mpox patients suggests a limited potentials for using CCGs as biomarkers or therapeutic targets in patient management. Future studies with larger Clades I and II cohorts, followed over time, are needed to discriminate between clade‐specific CCG expression and underlying comorbidities and complications.

## Author Contributions

Eugene Bangwen, Nicole Berens‐Riha, Wim Adriaensen, and Laurens Liesenborghs conceptualized the study. Eugene Bangwen, Nicole Berens‐Riha, Ann Ceulemans, Thao‐Thy Pham, and Marjan Van Esbroeck managed the samples and Wim Adriaensen coordinated the laboratory analyses at the Institute of Tropical Medicine. Eugene Bangwen, Nicole Berens‐Riha, and Ann Ceulemans performed the testing at the Institute of Tropical Medicine. Eugene Bangwen, Nicole Berens‐Riha, and Nicky de Vrij analyzed the data. Eugene Bangwen and Nicole Berens‐Riha wrote the first draft of the manuscript, with review and editing by Nicky de Vrij, Wim Adriaensen, and Laurens Liesenborghs. Laurens Liesenborghs secured the funding. All authors reviewed and approved the final version of the manuscript.

## Ethics Statement

The study was approved by the Institutional Review Board of the Institute of Tropical Medicine (ITM IRB) and the Ethics Committee of the University Hospital of Antwerp (EC UZA) reference numbers 1641/22, d.d. 10/31/2022 and 56/1818 d.d. 10/8/2020, respectively. Ethical approval for the use of serum samples from healthy volunteers was obtained from a separate study approved by ITM IRB and EC UZA with an open consent for further use of their biological samples for further research (1091/16 d.d. 06/29/2022 and 3117 d.d. 06/09/2022, respectively).

## Consent

Only samples from mpox patients who had provided a written informed consent for the secondary use of their samples for future research purposes were selected. The 10 longitudinal pre‐outbreak samples from PLWH were from regular outpatient department patients who had agreed on biobanking of their samples for future research. Only participants who had agreed to the future use of their samples were included.

## Conflicts of Interest

L.L. has received institutional consultancy fees from BioNtech and institutional research funding from Sanofi; both not relevant for this work. All other authors declare no competing interests.

## Supporting information

R1_Supp_Figure_1.png. Elevated CCGs in HIV‐negative mpox patients compared to respective controls. Secondary finding revealed a significant decrease in several CCG values during mpox in PLWH

R1_Supp_Figure_2.png. Decreased CCG values in PLWH mpox patients compared to respective controls. In HIV‐negative patients and controls, CCGs showed significant values either increasing or decreasing

Suppl. Table 1. All cytokines in all 136 samples from 126 participants (26 healthy controls, 10 mpox‐negative PLWH controls and 100 mpox HIV‐negative and PLWH patients). In case of significant interaction due to HIV, the analysis was split up in the two strata. We corrected for multiple testing using the Benjamini–Hochberg approach with a false discovery rate of 0.1.

## Data Availability

Deidentified data used in this study will be made available upon reasonable request.
