# Supplementary figures and images for: No Distinct Cytokine, Chemokine, and Growth Factor Blood Profile Associated With Monkeypox Virus Clade IIb Infected Patients
Source: J Med Virol. 2025 Mar 29;97(4):e70320. doi: 10.1002/jmv.70320 (PMC11954153; doi:10.1002/jmv.70320)

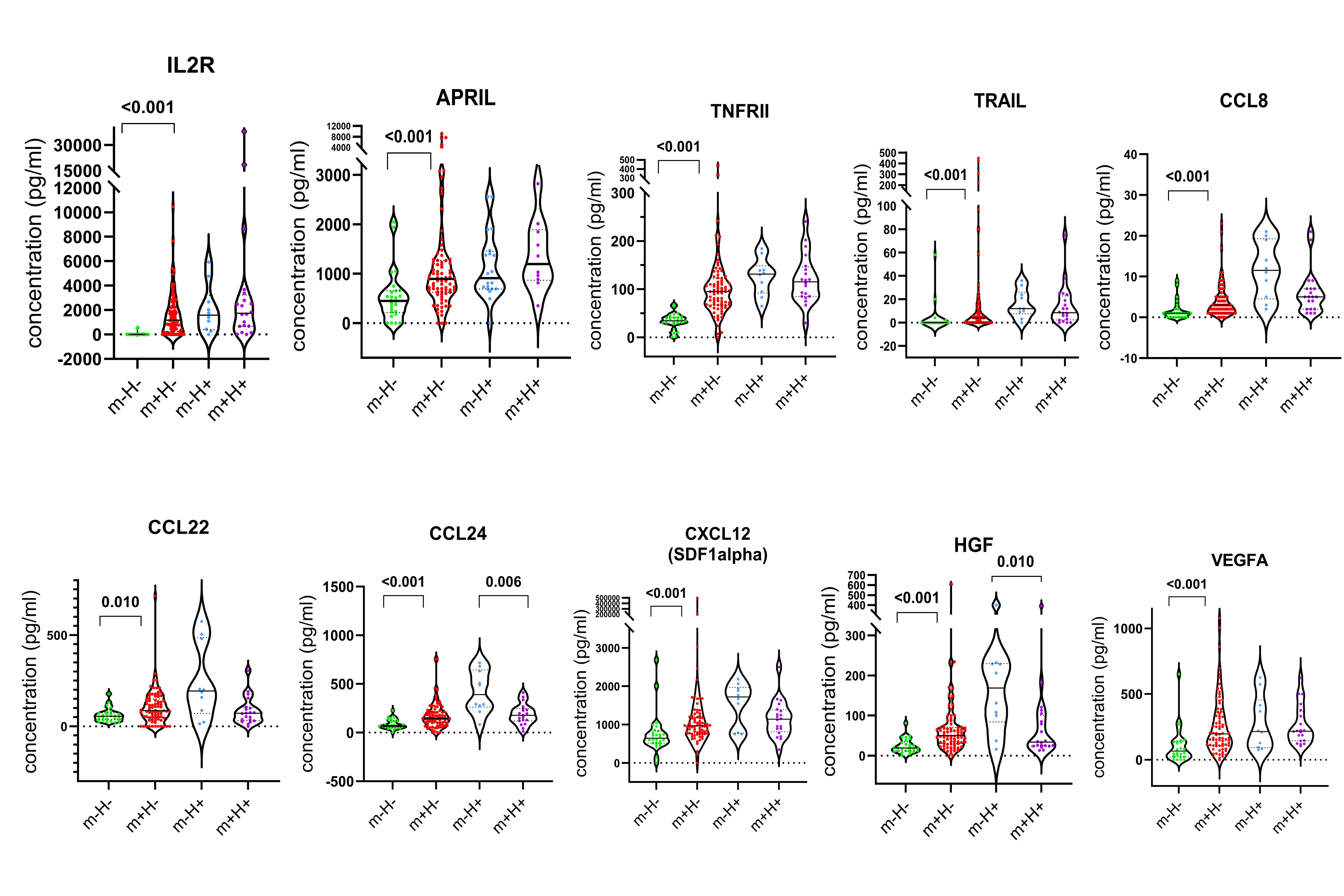

Supplement: Supplementary file 1 — R1_Supp_Figure_1.png. Elevated CCGs in HIV‐negative mpox patients compared to respective controls. Secondary finding revealed a significant decrease in several CCG values during mpox in PLWH [file JMV-97-e70320-s003.png]

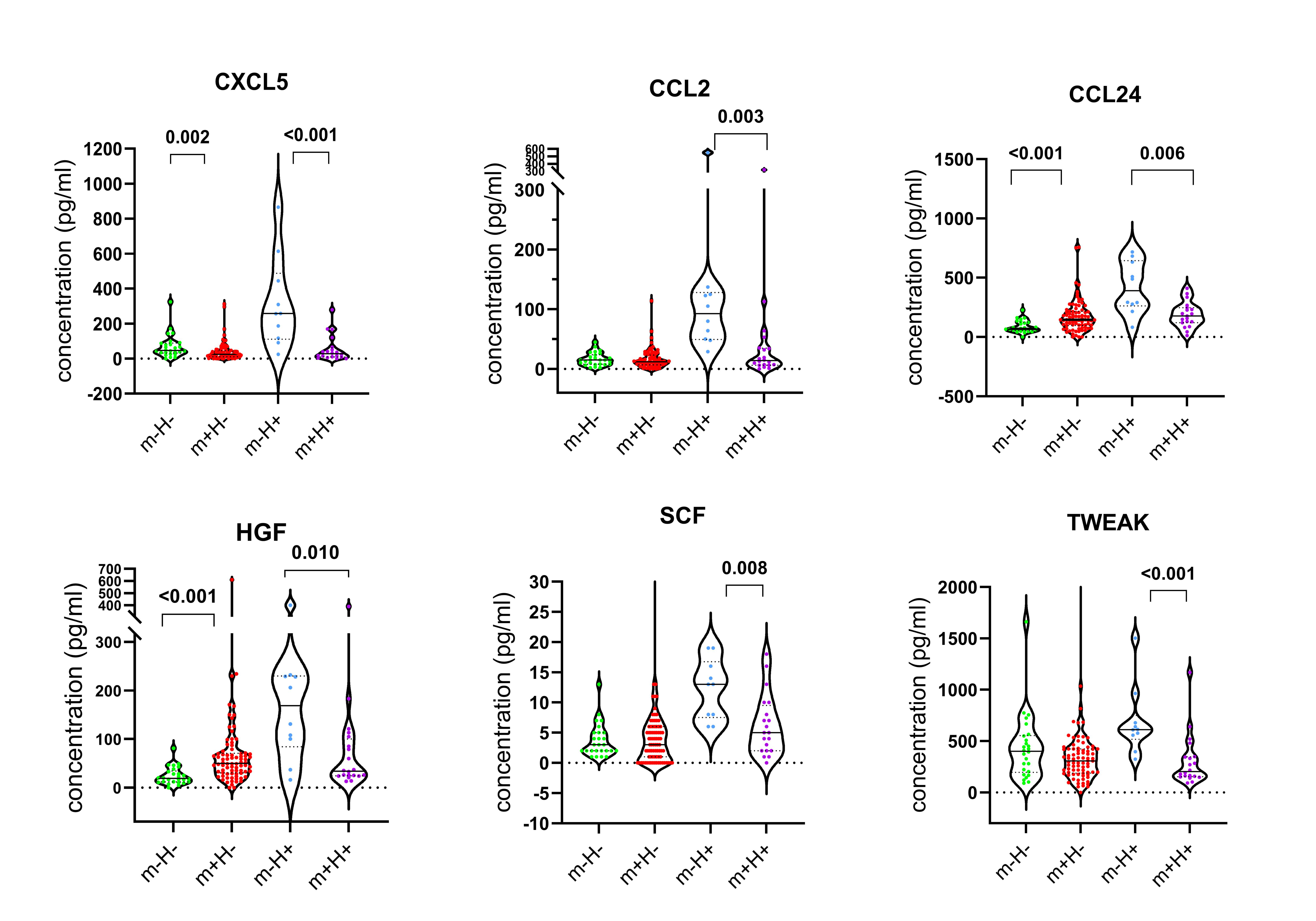

Supplement: Supplementary file 2 — R1_Supp_Figure_2.png. Decreased CCG values in PLWH mpox patients compared to respective controls. In HIV‐negative patients and controls, CCGs showed significant values either increasing or decreasing [file JMV-97-e70320-s001.png]
