# Supplementary material for: No Distinct Cytokine, Chemokine, and Growth Factor Blood Profile Associated With Monkeypox Virus Clade IIb Infected Patients
Source: J Med Virol. 2025 Mar 29;97(4):e70320. doi: 10.1002/jmv.70320 (PMC11954153; doi:10.1002/jmv.70320)
Supplement: Supplementary file 3 — Suppl. Table 1. All cytokines in all 136 samples from 126 participants (26 healthy controls, 10 mpox‐negative PLWH controls and 100 mpox HIV‐negative and PLWH patients). In case of significant interaction due to HIV, the analysis was split up in the two strata. We corrected for multiple testing using the Benjamini–Hochberg approach with a false discovery rate of 0.1. [file JMV-97-e70320-s002.docx]

**Suppl. table 1. All cytokines in all 136 samples from 126 participants (26 healthy controls, 10 mpox-negative PLWH controls and 100 mpox HIV-negative and PLWH patients). In case of significant interaction due to HIV, the analysis was split up in the two strata. We corrected for multiple testing using the Benjamini-Hochberg approach with a false discovery rate of 0.1.**

| Cytokine  (normal range in pg/mL if estab-lished) | Overall  (n=136) | | | | Inter- action with HIV | HIV-negative  (n=106) | | | | PLWH  (n=30 samples from 20 patients; ^b^ from 10 out of 20 longitudinal sampling: before and during mpox -> within-patient control) | | | |
| --- | --- | --- | --- | --- | --- | --- | --- | --- | --- | --- | --- | --- | --- |
|  | Overall | Mpox neg  (n=36) | Mpox pos  (n=100) |  |  | Overall | Mpox neg  (n=26) | Mpox pos  (n=80) |  | Overall | Mpox neg  (n=10) | Mpox pos  (n=20) |  |
|  | Median (IQR) | | | p-value^a^ | p-value^b^ | Median (IQR) | | | p-value^a^ | Median (IQR) | | | p-value^a^ |
| April | 809  (531; 1292) |  |  |  | *0.008* | 736  (446; 1211) | 450  (215; 655) | 901  (627; 1278) | **<0.001** | 1039  (728;  1860) | 1197  (868;  1889) | 1025  (691;  1788) | 0.291 |
| Baff | 0 (0; 0) | 0 (0; 0) | 0 (0; 0) | 0.812 | 0.428 |  |  |  |  |  |  |  |  |
| CXCL13  (26-507) | 76  (49; 124) |  |  |  | *0.040* | 68  (48; 115) | 54  (26; 79) | 80  (53; 124) | 0.020 | 99  (56; 149) | 53  (25;100) | 129  (83;199) | 0.003 |
| CD30  (25-110) | 263  (88; 577) | 46  (23;124) | 313  (177;736) | **<0.001** | 0.530 | 230  (59; 520) | 37  (21; 66) | 312  (155;702) | **<0.001** | 324  (165;647) | 242  (108;352) | 348  (250;897) | 0.059 |
| CD40L  (<118) | 21  (0; 60) | 2  (0; 26) | 23  (0; 59) | 0.053 | 0.388 |  |  |  |  |  |  |  |  |
| CXCL5 | 35  (13; 72) |  |  |  | *<0.001* | 34  (13; 61) | 47  (32; 89) | 25  (11; 46) | **0.002** | 58  (16; 205) | 258  (111;487) | 34  (10; 82) | **<0.001** |
| CCL11  (2-39) | 24  (15; 35) | 33  (21; 56) | 23  (15; 33) | **0.003** | 0.560 |  |  |  |  |  |  |  |  |
| CCL24 | 143  (76; 227) |  |  |  | *<0.001* | 68  (136; 207) | 69  (54; 126) | 146  (97; 216) | **<0.001** | 127  (222;376) | 391  (263;645) | 168  (109;262) | **0.006** |
| CCL26 | 0 (0; 0) | 0 (0; 0) | 0 (0; 0) | 0 (0;0) | 0.281 |  |  |  |  |  |  |  |  |
| FGF | 0 (0; 0) | 0 (0; 0) | 0 (0; 0) | 0.373 | 0.575 |  |  |  |  |  |  |  |  |
| CX3CL1 | 0 (0; 3) | 0 (0; 0) | 0 (0; 3) | 0.098 | 0.673 |  |  |  |  |  |  |  |  |
| G-CSF | 0 (0; 0) | 0 (0; 0) | 0 (0; 0) | 0.781 | 0.896 |  |  |  |  |  |  |  |  |
| GM-CSF  (3-122) | 0  (0; 0) | 0  (0; 0) | 0  (0; 0) | 0.835 | 0.621 |  |  |  |  |  |  |  |  |
| CXCL1 | 0 (0; 0) | 0 (0; 0) | 0 (0; 0) | 0.943 | 0.936 |  |  |  |  |  |  |  |  |
| HGF | 44  (23; 78) |  |  |  | *<0.001* | 43  (20; 67) | 19  (12; 34) | 49  (30; 71) | **<0.001** | 70  (26; 144) | 169  (84; 230) | 34  (24; 100) | **0.010** |
| CXCL11 | 18  (9; 34) | 11  (6; 15) | 20  (11; 38) | **0.002** | 0.825 |  |  |  |  |  |  |  |  |
| IFN-α  (3-63) | 0  (0; 0) | 0  (0; 0) | 0  (0; 0) | 0.296 | 0.693 |  |  |  |  |  |  |  |  |
| IFN-γ  (0-124) | 1  (0; 5) | 0  (0;4) | 1  (0;5) | 0.246 | 0.668 |  |  |  |  |  |  |  |  |
| IL-1α  (0.5-1.4) | 0  (0; 0) | 0  (0; 0) | 0  (0; 0) | 0.531 | 0.057 |  |  |  |  |  |  |  |  |
| IL-1β  (<0.7) | 0  (0; 0) | 0  (0; 0) | 0  (0; 0) | 0.610 | 0.673 |  |  |  |  |  |  |  |  |
| IL-2  (0-90) | 0  (0; 0) | 0  (0; 0) | 0  (0; 0) | 0.838 | 0.721 |  |  |  |  |  |  |  |  |
| IL-2R  (28-594) | 837  (0; 2235) | 0  (0; 475) | 1281  (330; 2503) | **<0.001** | 0.590 | 669  (0; 1806) | 0  (0; 0) | 1145  (252; 2287) | **<0.001** | 1782  (622;  3217) | 1583  (394;  3194) | 2082  (697;  3328) | 0.628 |
| IL3  (13-170) | 0  (0; 0) | 0  (0; 0) | 0  (0; 0) | 0.245 | 0.502 |  |  |  |  |  |  |  |  |
| IL4  (0-3) | 0  (0; 0) | 0  (0; 0) | 0  (0; 0) | 0.310 | 0.108 |  |  |  |  |  |  |  |  |
| IL5  (0-7) | 0  (0; 0) | 0  (0; 0) | 0  (0; 0) | 0.600 | 0.717 |  |  |  |  |  |  |  |  |
| IL6  (0-9) | 0  (0; 0) | 0  (0; 0) | 0  (0; 0) | 0.687 | 0.788 |  |  |  |  |  |  |  |  |
| IL7  (0- 14) | 0  (0; 1) |  |  |  | **<0.001** | 0  (0; 1) | 0  (0; 0) | 0  (0; 1) | **0.004** | 1  (0; 2) | 3  (1; 4) | 0  (0; 1) | **<0.001** |
| IL8  (0-116) | 0  (0; 0) | 0  (0; 0) | 0  (0; 0) | 0.247 | 0.916 |  |  |  |  |  |  |  |  |
| IL9  (0-500) | 0  (0; 0) | 0  (0; 0) | 0  (0; 0) | 0.247 | 0.225 |  |  |  |  |  |  |  |  |
| IL10  (0-2) | 0  (0; 0) | 0  (0; 0) | 0  (0; 0) | 1.000 | 1.000 |  |  |  |  |  |  |  |  |
| IL12  (0-6) | 0  (0; 0) | 0  (0; 0) | 0  (0; 0) | 0.584 | 0.215 |  |  |  |  |  |  |  |  |
| IL13  (0-9) | 0  (0; 0) | 0  (0; 0) | 0  (0; 0) | 0.610 | 0.191 |  |  |  |  |  |  |  |  |
| IL15  (0-5) | 0  (0; 0) | 0  (0; 0) | 0  (0; 0) | 0.536 | 0.674 |  |  |  |  |  |  |  |  |
| IL16 | 129  (71; 212) | 127  (66;156) | 137  (80;241) | 0.089 | 0.055 |  |  |  |  |  |  |  |  |
| IL17  (0-31) | 0  (0; 0) | 0  (0; 0) | 0  (0; 0) | 0  (0; 0) | 0.403 |  |  |  |  |  |  |  |  |
| IL18  (9-812) | 15  (6; 23) | 5  (3; 9) | 19  (10; 26) | **<0.001** | 0.290 |  |  |  |  |  |  |  |  |
| IL20 | 0 (0; 4) | 0 (0; 5) | 0 (0; 4) | 0.589 | 0.444 |  |  |  |  |  |  |  |  |
| IL21 | 0 (0; 0) | 0 (0; 0) | 0 (0; 0) | 0.190 | 0.413 |  |  |  |  |  |  |  |  |
| IL22 | 0 (0; 0) | 0 (0; 0) | 0 (0; 0) | 0.283 | 0.593 |  |  |  |  |  |  |  |  |
| IL23 | 0 (0; 0) | 0 (0; 0) | 0 (0; 0) | 0.469 | 0.557 |  |  |  |  |  |  |  |  |
| IL27 | 0 (0; 0) | 0 (0; 0) | 0 (0; 0) | 0.433 | 0.564 |  |  |  |  |  |  |  |  |
| IL31 | 0 (0; 0) | 0 (0; 0) | 0 (0; 0) | 0.469 | 0.696 |  |  |  |  |  |  |  |  |
| CXCL10  (6-637) | 16  (8; 32) | 8  (5; 13) | 19  (12; 35) | **<0.001** | 0.184 |  |  |  |  |  |  |  |  |
| LIF  (4-55) | 2 (0; 3) | 1 (0; 4) | 2 (0; 3) | 0.519 | 0.650 |  |  |  |  |  |  |  |  |
| M-CSF  (6-208) | 0 (0; 0) | 0 (0; 0) | 0 (0; 0) | 0.247 | 0.703 |  |  |  |  |  |  |  |  |
| CCL2  (2-48) | 713  (7; 28) |  |  |  | *0.035* | 13  (7; 23) | 15  (8; 24) | 12  (6; 24) | 0.411 | 33  (9; 107) | 50  (93; 128) | 14  (6; 48) | **0.003** |
| CCL8 | 3 (1; 6) |  |  |  | ***<****0.001* | 2 (1; 5) | 1 (0; 2) | 3 (1; 5) | **<0.001** | 6 (3; 11) | 12 (5; 20) | 5 (2; 9) | 0.036 |
| CCL7 | 0 (0;3) | 0 (0; 3) | 0 (0; 3) | 0.965 | 0.998 |  |  |  |  |  |  |  |  |
| CCL22 | 79  (40; 140) |  |  |  | *<0.001* | 78  (41, 134) | 55  (38; 74) | 86  (48; 150) | **0.010** | 81  (34; 188) | 194  (71, 486) | 72  (31; 104) | 0.026 |
| MIF | 34  (24; 46) | 23  (20; 35) | 38  (27; 54) | **<0.001** | 0.807 |  |  |  |  |  |  |  |  |
| CXCL9 | 2  (0; 40) | 0  (0; 0) | 17  (0; 48) | **<0.001** | 0.691 |  |  |  |  |  |  |  |  |
| CCL3  (<2.0) | 4  (2; 9) | 2  (0; 7) | 5  (2; 9) | **0.028** | 0.623 |  |  |  |  |  |  |  |  |
| CCL4  (1.7-47.0) | 49  (24; 70) | 25  (4; 47) | 58  (32; 76) | **<0.001** | 0.267 |  |  |  |  |  |  |  |  |
| CCL20 | 1 (0; 5) | 0 (0;3) | 2 (0; 6) | 0.026 | 0.096 |  |  |  |  |  |  |  |  |
| MMP-1 | 222  (137; 426) |  |  |  | 0.121 | 213  (125; 394) | 189  (109;256) | 226  (127;419) | 0.105 | 345  (176;736 | 640  (336;782) | 231  (167; 677) | 0.075 |
| NGF-β  (<1.1) | 0 (0; 0) | 0 (0; 0) | 0 (0; 0) | 0.405 | 0.416 |  |  |  |  |  |  |  |  |
| SCF  (16-837) | 4 (2; 7) |  |  |  | *0.004* | 3 (1; 6) | 3 (2;5) | 3 (0; 6) | 0.625 | 8 (4; 13) | 13 (8; 17) | 6 (2; 10) | **0.008** |
| CXCL12/  SDF1α  (8-92) | 949  (734;  1260) |  |  |  | *0.011* | 896  (706; 1173) | 644  (516; 836) | 967  (772; 1221) | **<0.001** | 1178  (784; 1665) | 1720  (777; 1968) | 1118  (810;  1331) | 0.053 |
| TNF-α  (0-98) | 0 (0; 0) | 0 (0; 0) | 0 (0; 0) | 0.780 | 0.437 |  |  |  |  |  |  |  |  |
| TNF-β  (0-13) | 0 (0; 0) | 0 (0; 0) | 0 (0; 0) | 0.469 | 0.647 |  |  |  |  |  |  |  |  |
| TNF-RII | 85  (50;120) |  |  |  | *0.003* | 74  (42; 110) | 35  (30; 42) | 67  (95; 122) | **<0.001** | 120  (85; 143) | 92  (132;149) | 113  (85; 146) | 0.567 |
| TRAIL  (8-272) | 3 (0; 12) |  |  |  | 0.386 | 1 (0; 8) | 0 (0; 0) | 4 (0; 11) | **0.002** | 10 (3; 22) | 12 (8; 26) | 8 (1; 18) | 0.402 |
| TSLP | 1 (0;3) | 0 (0; 5) | 0 (0; 3) | 0.635 | 0.448 |  |  |  |  |  |  |  |  |
| TWEAK | 332  (193; 480) |  |  |  | *0.031* | 329  (193; 443) | 401  (196; 554) | 307  (188; 423) | 0.126 | 338  (163; 610) | 612  (518; 750) | 204  (153; 348) | **<0.001** |
| VEGFA  (0-9) | 166  (97; 334) |  |  |  | 0.056 | 153  (79; 318) | 66  (22; 141) | 197  (110; 360) | **<0.001** | 221  (140; 417) | 215  (92; 455) | 221  (156; 413) | 0.598 |

^a^ Mann-Whitney test, raw p-values are presented, in bold significant p-values after Benjamini-Hochberg correction for multiple comparisons

^b^ Analyzed by a regression model testing for interaction. If the interaction term was significant (p-value < 0.05) or the Mantel-Haenszel test showed very different outcomes, separate p-values are presented. Mann Whitney U test was used to compare between the different groups.
